# Adolescent sports participation and school adjustment: the chain-mediation roles of peer relationships and depressive symptoms

**DOI:** 10.3389/fpubh.2026.1884531

**Published:** 2026-07-13

**Authors:** Chenchen Liu, Xinxin Lai, Yan Gao, Yuke Yang, Liangyu Zhao

**Affiliations:** 1 School of Medical Information Engineering, Jining Medical University, Jining, China; 2 School of Physical Education, Shandong University, Jinan, China; 3 Department of Biomedical Engineering, The Hong Kong Polytechnic University, Hong Kong, Hong Kong SAR, China

**Keywords:** adolescents, depressive symptoms, peer relationships, school adjustment, sports participation

## Abstract

**Objective:**

This study examined the relationship between adolescent sports participation and school adjustment and analyzed the serial mediating effects of peer relationships and depressive symptoms.

**Methods:**

The study sample comprised 8,096 adolescent participants. Assessments were conducted using the Physical Activity Questionnaire for Children (PAQ-C), the School Social Behavior Scale (SSBS), the Quality of Life Scale for Children and Adolescents (QLSCA), and the Symptom Checklist-90 (SCL-90). The hypothesized mediation effects were tested using the SPSS 26.0 macro PROCESS 4.5 with bias-corrected bootstrapping (5,000 resamples) to assess the significance of the indirect effects.

**Results:**

Group comparisons showed that boys scored higher than girls on sports participation, school adjustment and reported lower depressive symptoms scores (*p* < 0.001), while no significant gender difference emerged for peer relationships. No significant age differences were observed for sports participation, school adjustment, or peer relationships (all *p* > 0.05), but depressive symptoms increased significantly with age (*p* < 0.01), with the highest levels at age 19. Only children scored higher on sports participation and school adjustment and lower on depressive symptoms, but lower on peer relationships, than non-only children (*p* < 0.001). Students in remote areas showed lower school adjustment and higher depressive symptoms scores than those in several non-remote groups (*p* < 0.001). Sports participation was positively correlated with school adjustment (*r* = 0.260, *p* < 0.001) and peer relationships (*r* = 0.201, *p* < 0.001) and negatively correlated with depressive symptoms (*r* = −0.166, *p* < 0.001). Peer relationships were positively correlated with school adjustment (*r* = 0.344, *p* < 0.001) and negatively correlated with depressive symptoms (*r* = −0.362, *p* < 0.001). Depressive symptoms were negatively correlated with school adjustment (*r* = −0.226, p < 0.001). Peer relationships (effect = 1.76, 95% CI [1.50, 2.03]) and depressive symptoms (effect = 0.23, 95% CI [0.14, 0.33]) each mediated the association between sports participation and school adjustment. They also formed a small but statistically significant serial mediation effect pathway (effect = 0.20, 95% CI [0.14, 0.28]).

**Conclusion:**

This study further clarifies the relationship between adolescent sports participation and school adjustment, and emphasizes the chain mediating role of peer relationships and depressive symptoms. These findings provide empirical evidence for the educational value of adolescent sports participation and offer practical implications for optimizing school physical education to enhance school adjustment and promote mental health.

## 1 Introduction

Adolescence is a critical transitional and accelerated period in the physical and psychological development of the individual. During this period, mental health issues have garnered increasing scholarly attention. Approximately 20% of teenagers worldwide are affected by depressive symptoms (1), while the overall prevalence of depressive symptoms among Chinese teenagers is 22.2% (2). As the primary place for learning and living, schools have an important role in shaping their development. Within the school context, physical education is not only a curriculum for improving physical fitness and motor skills, but also an important educational setting for cultivating cooperation, emotional regulation, rule awareness, and peer interaction. Well-organized physical education teaching can provide structured opportunities for adolescents to participate in sports, build supportive peer relationships, and develop positive adaptive behaviors. Adolescents’ adaptability has become a crucial quality during the growth process of teenagers. Studies have shown that sports participation has a positive impact on school adjustment (3). However, the strength of this impact varies significantly depending on the individual’s level of physical activity (4) and gender (5). Furthermore, sports participation can enhance adolescents’ learning concentration, self-management skills, and interpersonal abilities (6). Therefore, this study proposes:

Hypothesis 1: Sports participation is positively correlated with school adjustment.

Peer relationships, as an important part of socialization during adolescent development (7), have a significant influence on adolescents’ self-awareness (8) and emotional regulation (9). The effect of sports participation on teenagers’ peer relationships is influenced by exercise intensity (10) and skill performance (11). At the same time, the quality of peer friendships (12) and peer attachment (13) can positively predict children’s adaptive abilities, indicating that peer relationships can facilitate adolescents’ better social and school adjustment. This study proposes:

Hypothesis 2: Peer relationships play a partial mediating role between sports participation and school adjustment.

However, current research on depressive symptoms mainly focuses on different populations (14), gender differences (15), and the internal connections with related variables (16, 17). As an effective method of improving depressive symptoms, an increasing number of studies have shown that sports participation can significantly reduce the depression of adolescents (18, 19), and shows significant effects in different types of sports (20). Moreover, depression is strongly correlated with rule adaptation, interpersonal relationships, and academic adaptation in school adjustment (21). Therefore, this study proposes:

Hypothesis 3: Depressive symptoms shows a statistically significant indirect effect in the relationship between sports participation and school adjustment.

This study adopts Self-Determination Theory as the core theoretical framework to explain the mechanism through which sports participation is associated with school adjustment. This theory emphasizes the impact of satisfying autonomy, competence, and relatedness on an individual’s motivation (22). In this context, sports participation, as a supportive behavioral context, provides opportunities for social interaction for teenagers, effectively meeting their need for relatedness, thereby promoting positive peer relationships; this high-quality peer relationship further consolidates their relatedness and enhances their sense of competence and autonomy, thereby reducing depressive symptoms; ultimately, students with fewer depressive symptoms can engage in school life with higher intrinsic motivation and emotional engagement, leading to better school adjustment in terms of academic, social, and emotional aspects. Thus, this study proposes:

Hypothesis 4: Peer relationships and depressive symptoms play a chain mediating role in the relationship between sports participation and school adjustment.

However, several limitations remain in the existing literature. Although previous studies have examined the direct relationship between sports participation and school adjustment or have separately tested individual mediators such as peer relationships and depressive symptoms, limited research has systematically investigated the serial mediating pathway linking sports participation to school adjustment through peer relationships and subsequent depressive symptoms. This gap restricts a more comprehensive understanding of the psychosocial mechanisms underlying adolescent school adjustment. In addition, many prior studies have relied on relatively small samples, limiting the generalizability and practical applicability of their findings. Therefore, it remains unclear whether sports participation improves school adjustment not only directly but also indirectly through the sequential enhancement of peer relationships and reduction of depressive symptoms.

The core contributions of this study are threefold. First, based on a large sample of 8,096 adolescents, it enhances statistical power and robustness of the findings. Second, it systematically tests the serial mediating pathway of “sports participation → peer relationships → depressive symptoms → school adjustment” for the first time, addressing the gap in previous studies that focused mainly on single mediators. Third, it systematically compares differences across gender, residence, and only-child status on these variables, providing demographic evidence for targeted interventions.

This study focuses on the adolescent population to examine the relationship between sports participation and school adjustment. It further investigates the partial mediating roles and chain mediating effect of peer relationships and depressive symptoms. From the perspective of physical education, this study may help clarify how school-based sports participation contributes to adolescents’ social adaptation and psychological development, thereby providing evidence for optimizing physical education teaching content, organizing diversified sports activities, and improving the educational function of school sports. The findings aim to provide a more scientific and comprehensive theoretical foundation and, more importantly, to offer concrete, actionable implications for school-based mental health interventions and physical education curriculum design, there by promoting the healthy development of adolescents.

## 2 Materials and methods

### 2.1 Study design

A questionnaire-based survey employing Probability Proportional to Size (PPS) sampling was conducted among adolescent students in Shandong Province from October 2024 to March 2025. In this procedure, selection probabilities were allocated to primary sampling units commensurate with their size to minimize sampling error and guarantee the representativeness and objectivity of the final sample. Data were collected using an electronic questionnaire. Students were escorted by trained classroom advisors to the computer lab during self-study periods, where they completed and submitted the questionnaire online. The response time averaged 20–25 min per participant. Investigators remained on site to answer questions and ensure standardized data collection. A total of 8,500 questionnaires were distributed and collected. After eliminating the invalid responses, 8,096 valid questionnaires were finally obtained. The following criteria were used to exclude invalid questionnaires: (1) completion time less than 5 min (the mean completion time in the pilot test was 20 min); (2) selecting the same response option for more than 10 consecutive items; (3) more than 20% missing responses on any key variable scale; (4) obvious patterned responses. After applying these criteria, a total of 8,096 valid questionnaires were retained, yielding an effective response rate of 95.2%. All participants and their guardians had given informed consent, and it was approved by the Ethics Committee of Shandong University (number: 20180517). All data collected in this study are stored in the Population Health Data Archive (PHDA), specifically within the Database of Youth Health (DYH).

### 2.2 Measurement

#### 2.2.1 Sports participation

The Physical Activity Questionnaire for Children (PAQ-C) was employed to assess the level of sports participation (23). The present study utilized a Chinese revised version of the PAQ-C, which was adapted and validated for use with Chinese adolescent populations (24). This questionnaire consists of nine items and uses a 5-point Likert scale. The total score is the average of the first eight items, which measure the frequency and intensity of sports participation in various contexts [e.g., “In the past 7 days, did you engage in any of the following physical activities? If yes, how many times?” (1 = never to 5 = more than 7 times) and “In the past 7 days, how often did you engage in vigorous physical activity during PE class?” (1 = no PE class to 5 = always)]. A higher total score indicates a higher level of sports participation. This questionnaire has good reliability and validity (Cronbach’s *α* = 0.92). Although the PAQ-C was originally designed to measure general physical activity, this study operationalizes it as sports participation (i.e., structured, voluntary activities in sports contexts). Accordingly, we use “sports participation” throughout, reserving “physical activity” only for references to other studies.

#### 2.2.2 School adjustment

The School Social Behavior Scale (SSBS) was used to assess students’ adaptive behaviors in the school context (25). This 65-item scale is divided into social competence and antisocial behavior. It employs a 5-point Likert scale, with scores indicating the frequency of each behavior (e.g., “Most of the time I can cooperate with other classmates” and “I can smoothly transition between different classroom activities,” rated from 1 = never to 5 = always). The antisocial behavior dimension adopts a negative scoring method, meaning that higher scores on this subscale reflect poorer school adjustment. Because the Antisocial Behavior dimension is negatively keyed (higher raw scores indicate poorer adjustment), each item on that subscale was first reversed so that higher values uniformly represent better adjustment. The sum of these two transformed subscale scores constitutes the overall School Adjustment score, with higher totals reflecting more positive school adaptation (26). In the current study, this composite score demonstrated excellent internal consistency (Cronbach’s *α* = 0.94).

#### 2.2.3 Peer relationship

The Quality of Life Scale for Children and Adolescents (QLSCA) was used to assess peer relationship (27). This scale is commonly used to measure the learning and living conditions, physical, psychological, social functions, and living environment of students aged 7–18. It comprises 13 dimensions grouped under four factors and employs a 4-point Likert scale. To assess participants’ peer relationships, we used the Peer Relationship subscale of the QLSCA. This subscale consists of 5 items [e.g., “Do you feel that your classmates are friendly to you?” (1 = not friendly, 4 = very friendly) and “Do your friends care about you?” (1 = not at all, 4 = very much). Higher total scores indicate a better overall peer relationship (Cronbach’s *ɑ* = 0.91)].

#### 2.2.4 Depressive symptoms

The Symptom Checklist 90 (SCL-90) was used to assess the severity of the 9 dimensions, with a total of 90 questions (28). The depressive symptoms was used to reflect the broad concept associated with clinical depressive symptoms clusters. Items are rated on a 5-point Likert scale, with higher scores indicating greater severity of depressive symptoms (e.g., “Loss of sexual interest or pleasure” and “Thoughts of ending one’s life,” rated from 1 = not at all to 5 = extremely severe.). In this study, the depressive symptoms subscale demonstrated good internal consistency (Cronbach’s *ɑ* = 0.95).

### 2.3 Statistical analysis

Statistical analyses were performed with Excel and SPSS 26.0. This included descriptive statistics, independent-samples *t*-tests, and Pearson correlations. The PROCESS 4.5 macro program (Model 6) for SPSS 26.0 was employed to verify and analyze the mediating model, and the size of the mediating effect and its confidence interval were estimated using a 95% confidence interval. Statistical significance was set at *p* < 0.05.

## 3 Results

### 3.1 Comparison of scores for different population characteristics

In terms of gender, boys scored higher than girls on sports participation, school adjustment and reported lower depressive symptoms scores (*p* < 0.001), while no significant gender difference emerged for peer relationships. No significant age differences were found in sports participation, school adjustment, or peer relationship scores (all *p* > 0.05). However, a significant age effect was observed for depressive symptoms (*p* < 0.01). *Post-hoc* comparisons indicated that depressive symptoms tended to increase with age. The highest mean depressive symptoms score was observed among 19-year-old students, followed by 18-year-old and 15-year-old. Residential differences revealed that students living in other cities scored the highest in sports participation, school adjustment, and peer relationship, while students living in urban–rural fringe areas had the highest scores for depressive symptoms (*p* < 0.001). Compared with non-only children, only children scored significantly higher on sports participation and school adjustment, but lower on peer relationships. Notably, they also reported significantly lower levels of depressive symptoms (*p* < 0.001). The results are shown in Table 1.

**Table 1:** Comparison of score differences for each scale based on different demographic (*N* = 8,096).

Variable	Group (%)	Sports participation	School adjustment	Peer relationship	Depressive symptoms
Gender	Male (47%)	2.26 ± 0.76	169.17 ± 23.18	15.40 ± 3.33	19.06 ± 8.10
	Female (53%)	2.01 ± 0.66	166.50 ± 20.65	15.37 ± 3.10	20.58 ± 8.71
	F	5.45	5.45	0.33	−8.11
	*p*	<0.001	<0.001	0.743	<0.001
Age	14	2.09 ± 0.64	162.85 ± 20.85	15.28 ± 2.79	18.32 ± 7.77
	15	2.12 ± 0.70	168.61 ± 20.97	15.38 ± 3.35	20.00 ± 8.70
	16	2.13 ± 0.69	167.65 ± 21.42	15.44 ± 3.17	19.63 ± 8.24
	17	2.11 ± 0.73	167.84 ± 22.93	15.36 ± 3.11	19.79 ± 8.44
	18	2.14 ± 0.76	167.55 ± 21.69	15.34 ± 3.30	20.41 ± 8.50
	19	2.13 ± 0.75	166.71 ± 21.91	15.31 ± 3.20	21.31 ± 10.24
	F	0.27	2.02	0.31	3.41
	*p*	0.930	0.072	0.909	0.004
Residence	Central city area (47%)	2.14 ± 0.74	169.04 ± 21.99	15.52 ± 3.31	19.49 ± 8.80
	Remote areas (8%)	2.16 ± 0.71	166.73 ± 22.36	14.42 ± 3.69	21.55 ± 8.68
	Township (22%)	2.13 ± 0.69	167.19 ± 22.26	15.46 ± 3.00	19.97 ± 7.80
	Other counties (2%)	2.38 ± 0.78	170.95 ± 18.90	15.79 ± 3.25	19.86 ± 8.79
	Rural (21%)	2.06 ± 0.68	165.46 ± 21.30	15.30 ± 2.85	20.00 ± 8.16
	F	10.97	9.63	16.68	8.03
	*p*	<0.001	<0.001	<0.001	<0.001
Only-child	Yes	2.199 ± 0.741	169.44 ± 24.08	15.10 ± 3.71	19.34 ± 8.48
	No	2.093 ± 0.703	166.94 ± 20.74	15.52 ± 2.90	20.12 ± 8.44
	F	6.16	4.55	−5.14	−3.89
	*p*	<0.001	<0.001	<0.001	<0.001

### 3.2 Common method bias

To control for common method bias, this study used Harman’s single-factor test. The results showed that the first unrotated factor accounted for less than 40% of the variance, indicating that common method bias is not a serious concern in this study.

### 3.3 Correlation analysis between variables

Pearson correlation analysis was used to examine the relationships among sports participation, school adjustment, peer relationship, and depressive symptoms. The results are presented in Table 2. There was a significant positive correlation between sports participation and school adjustment, as well as peer relationship (*p* < 0.001), with correlation coefficients of 0.260 and 0.201, respectively. Sports participation demonstrated a significant negative correlation with depressive symptoms (*p* < 0.001), with a correlation coefficient of −0.166. Peer relationship showed a significantly positive correlation with school adjustment (*p* < 0.001, *r* = 0.344). Conversely, it was significantly negatively correlated with depressive symptoms (*p* < 0.001, *r* = −0.362). Depressive symptoms was significantly negatively correlated with school adjustment (*p* < 0.001, *r* = −0.226).

**Table 2:** Correlations between study variables (*N* = 8,096).

Item	Sports participation	School adjustment	Peer relationship	Depressive symptoms
Sports participation	1.00			
School adjustment	0.26***	1.00		
Peer relationship	0.20***	0.34***	1.00	
Depressive symptoms	−0.17***	−0.23***	−0.36***	1.00

*p < 0.05, **p < 0.01, ***p < 0.001. SP, sports participation; PR, peer relationship; DS, depressive symptoms.

### 3.4 Mediation analysis involving peer relationship and depressive symptoms

#### 3.4.1 Regression analysis of sports participation, school adjustment, peer relationship, and depressive symptoms

Hierarchical multiple regression was conducted to examine predictors of school adjustment (SA), with control variables (gender, age, residence, only-child status) entered first, followed sequentially by sports participation (PA), peer relationships (PR), and depressive symptoms (DS) (Table 3). The model fit improved significantly at each step: Model 1, including control variables and PA, explained 7.1% of the variance in SA (R^2^ = 0.071, *p* < 0.001). Adding peer relationships significantly improved the model fit (ΔR^2^ = 0.090, *p* < 0.001), resulting in Model 2 explaining 16.1% of the variance (*R*^2^ = 0.161, *p* < 0.001). The addition of depressive symptoms further improved the model fit (Δ*R*^2^ = 0.007, *p* < 0.001), with the full Model 3 explaining 16.9% of the variance (*R*^2^ = 0.169, *p* < 0.001). In the final model, sports participation (B = 5.606, *p* < 0.001) and peer relationships (*B* = 1.891, *p* < 0.001) emerged as significant positive predictors of SA, while depressive symptoms (*B* = −0.238, *p* < 0.001), residence (B = −0.498, *p* = 0.001), and only-child status (*B* = −2.007, *p* < 0.001) were significant negative predictors. Gender and age were not significant in any of the three models.

**Table 3:** Regression analysis of sports participation, peer relationships, depressive symptoms and school adjustment (*N* = 8,096).

Variables/Statistics	Model 1				Model 2				Model 3			
	B	SE	t	*p*	B	SE	t	*p*	B	SE	t	*p*
Constant	155.703	3.967	39.254	<0.001	129.258	3.874	33.363	<0.001	136.153	3.945	34.516	<0.001
sex	−0.598	0.483	−1.238	0.216	−0.859	0.459	−1.872	0.061	−0.587	0.458	−1.282	0.2
Age	−0.02	0.225	−0.089	0.929	−0.009	0.214	−0.042	0.967	0.048	0.213	0.227	0.821
Home	−0.616	0.154	−4.009	<0.001	−0.495	0.146	−3.39	0.001	−0.498	0.145	−3.422	0.001
Child	−1.068	0.524	−2.039	0.041	−2.223	0.499	−4.453	<0.001	−2.007	0.498	−4.033	<0.001
PA	7.795	0.333	23.431	<0.001	5.834	0.323	18.062	<0.001	5.606	0.323	17.366	<0.001
PR					2.111	0.072	29.501	<0.001	1.891	0.076	24.9	<0.001
DS									−0.238	0.028	−8.379	<0.001
R^2^	0.071				0.161				0.169			
Adj. R^2^	0.07				0.161				0.168			
F	*F* (5,8,090) = 123.765***				*F* (6,8,089) = 259.273***				*F* (7,8,088) = 234.164***			
△R^2^	0.071				0.09				0.007			
△F	F (5,8,090) = 123.765***				*F* (1,8,089) = 870.314***				*F* (1,8,088) = 70.202***			

Dependent variable = School adjustment. SP, sports participation; PR, peer relationship; DS, depressive symptoms. *p < 0.05, **p < 0.01, ***p < 0.001.

#### 3.4.2 Testing the mediating effect of peer relationship on depressive symptoms

**Figure 1:**
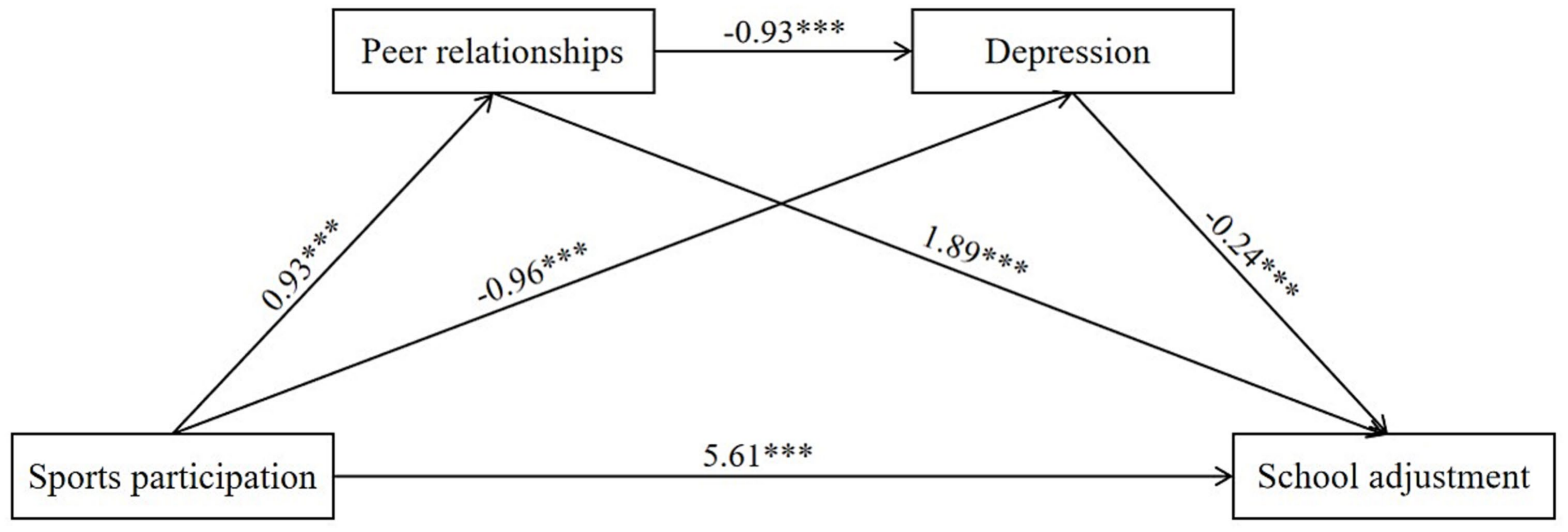
The chain mediating effect of peer relationships and depressive symptoms on sports participation and school adjustment.

After controlling for demographic variables, with sports participation as the independent variable, school adjustment as the dependent variable, and peer relationships and depressive symptoms as the mediating variables, a serial mediation analysis was conducted using SPSS 26.0 and PROCESS 4.5. The specific results of the chain mediating effect of peer relationship and depressive symptoms between sports participation and school adjustment were presented in Table 4 and Figure 1.

**Table 4:** The chain mediating effects of peer relationships and depressive symptoms (*N* = 8,096).

Model effect	Effect	Boot SE	Boot LLCI	Boot ULCI	Effect (%)
Total effect	7.79	0.33	7.14	8.45	100.00
Direct effect	5.61	0.32	4.97	6.24	71.92
Total indirect effect	2.19	0.13	1.51	2.02	28.08
X → M1 → Y	1.77	0.14	1.51	2.02	22.53
X → M2 → Y	0.23	0.05	0.14	0.33	2.93
X → M1 → M2 → Y	0.20	0.03	0.14	0.27	2.62

X = sport participation, M1 = peer relationship, M2 = depressive symptoms, Y = school adjustment. All indirect effects were controlled for gender, place of residence, and only child status.

The results showed that the effect value of the path from sports participation to school adjustment was 5.61 (95% CI [4.97, 6.24], *p* < 0.001), indicating that sports participation was directly associated with school adjustment. The indirect effect of sports participation on school adjustment through peer relationship was 1.76 (95% CI [1.50, 2.03], *p* < 0.001), indicating that sports participation had an indirectly associated with school adjustment through its positive relationship with peer relationships. The indirect effect of sports participation on school adjustment through depressive symptoms was 0.23 (95% CI [0.14, 0.33], *p* < 0.001). This suggests an indirect association between sports participation and school adjustment through reduced depressive symptoms. The specific indirect effect of sports participation on school adjustment through the serial mediation of peer relationship and depressive symptoms was 0.20 (95% CI [0.14, 0.28], *p* < 0.001). This indicated a small but significant indirect association between sports participation and school adjustment among adolescents through the chain mediating pathway of peer relationships and depressive symptoms.

## 4 Discussion

### 4.1 Differences in variables based on different demographic characteristics

In terms of gender, males scored higher than females in sports participation and school adjustment, and reported lower depressive symptoms scores, while no significant gender difference was observed for peer relationships. This finding is consistent with existing research (29–31). This might be related to the fact that males are more involved in sports participation and have relatively stable emotional regulation, while females tend to exhibit greater emotional sensitivity and are more susceptible to interpersonal and academic stressors (32, 33). Concurrently, societal gender role expectations may also play a significant role in shaping these gender differences in adaptation and mental health outcomes (34). At the regional level, the levels of sports participation and school adjustment among adolescents in remote areas were relatively low, while the scores for depressive symptoms were higher (35). This disparity may stem from the lack of relatively complete sports facilities and school psychological counseling services in remote areas (36, 37). Due to the lack of opportunities for sports participation and structured social interaction, adolescents in these areas have fewer ways to relieve stress, which may increase their vulnerability to depressive symptoms. In terms of family structure, only children score higher in sports participation and school adjustment, but lower in peer relationships and depressive symptoms. In this study, the advantages of only children in sports participation contrasts sharply with the commonly reported finding in Western studies that only children tend to be physically inactive (38). However, it is consistent with the findings of local Chinese research that only children have a higher participation rate in organized sports programs (26). This might be due to the resource compensation mechanism in the 4–2-1 family structure in China (four grandparents, two parents, one child) (39). The only child may receive more family resources and receive more attention, compensating for the lack of social interaction opportunities caused by the absence of sibling interaction (40). Meanwhile, they develop greater self-regulation skills developed from growing up without siblings. These protective factors likely outweigh the disadvantage of lower peer interaction quantity.

### 4.2 The positive impact of sports participation on school adjustment among adolescents

The results of this study showed that sports participation was significantly positively associated with school adjustment of adolescents. Although the correlation coefficient between sports participation and school adaptation is weak, the regression analysis still shows a significant positive predictive effect, indicating that there is a meaningful association between the variables. A meta-analysis showed that sports participation had a small to moderate positive effect on academic performance (41). Sports participation can strengthens overall fitness, enhances physical function (42), and boosts body immunity (43). These benefits may help adolescents meet the physical demands in school life. In addition, sports participation has been associated with enhanced cognitive functions, such as attention and memory (44, 45), which could in turn support academic achievement and strengthen adolescents’ confidence in adapting to school life. These physiological effects may contribute to better academic performance and could thereby enhance adolescents’ confidence and enthusiasm for adapting to school life. Moreover, adolescents who frequently engage in physical activities demonstrate better adaptability in school, including greater academic engagement (46) and fewer behavioral problems (47). The Self-Determination Theory provides a theoretical explanation for this relationship. Engaging in physical activities, as a supportive behavioral context, may satisfy the basic psychological needs of adolescents (48). The satisfaction of these psychological needs may enhance intrinsic motivation and may transfer to the school life domain, manifesting as greater learning engagement, more positive interactions between teachers and students, and better compliance with behavioral norms (49). Furthermore, sports participation has been associated with improvements in adolescents’ attention control and emotion regulation (50, 51). These improvements in executive functions may directly support their ability to cope with academic pressure and various challenges in the school environment (52). Therefore, these findings suggest that sports participation could be considered a potential resource for supporting adolescents’ school adjustment.

### 4.3 The mediating role of peer relationships between sport participation and school adjustment

This study found that peer relationships accounted for 22.53% of the total indirect effect between sports participation and school adjustment, suggesting that peer relationships may function as an important mediating factor. In other words, sports participation may not only directly associated with school adjustment, but could also be indirectly linked to it through more favorable the adolescents’ peer relationships. Sports fields and participation in sports offer adolescents frequent, fairly structured social settings (53). Adolescents need to cooperate, compete, communicate, and resolve conflicts with peers. These experiences may help build trust and friendship, and adolescents with better motor skills tend to be more accepted by their peers (54). Adolescents who participate in sports more frequently usually have a broader social network (55). These positive peer relationships are likely a source of emotional and instrumental support. More importantly, strong peer relationships may enhance their sense of belonging to school, potentially increasing their willingness to take part in school activities and follow school rules (56). In contrast, adolescents who lack peer support are more likely to feel isolated and experience psychological adjustment difficulties (57). Therefore, these findings suggest that sports participation may represent a potential pathway for promoting adolescent’ school adjustment, partly through its link with more positive peer relationships.

### 4.4 The mediating role of depressive symptoms between sport participation and school adjustment

Depressive symptoms was also found to be a significant mediator between sports participation and school adjustment, although the indirect effect through this pathway was relatively small. The protective effect of sports participation against depressive symptoms has been confirmed by a large number of studies. From a psychological perspective, sports participation provides manageable challenges and a sense of accomplishment, which may contribute to enhanced self-efficacy (58). Moreover, the social interactions that accompany sports participation may help reduce feelings of loneliness (59). In this study, the regression coefficient from sports participation to depressive symptoms was −0.96 (*p* < 0.001), indicating that higher levels of sports participation were associated with lower levels of depressive symptoms. Depressive symptoms has been consistently linked to poorer school adjustment. Depressive symptoms is a significant obstacle to school adjustment. Depression often involves cognitive difficulties such as poor concentration and memory decline (60), as well as behavioral issues like social withdrawal and loss of interest. These cognitive and behavioral difficulties are often associated with lower academic performance and reduced social interaction, which may in turn exacerbate school adjustment problems (61). The regression coefficient of depressive symptoms on school adjustment was −0.24 (*p* < 0.001), indicating a negative association between depressive symptoms and school adjustment in the current sample. Higher levels of depressive symptoms were associated with poorer school adjustment (62). Consequently, these findings suggest that sports participation may be indirectly associated with better school adjustment through its connection with lower depressive symptoms levels. Although the effect size of this pathway is modest, for adolescents who already exhibit mild depressive symptoms, exercise-based approaches might be considered a feasible complement to other interventions, as they may be relatively accessible and could indirectly benefit school adjustment.

### 4.5 The chain mediating role of peer relationships and depressive symptoms

A key finding of this study was the identification of a small but significant serial indirect pathway linking sports participation, peer relationships, depressive symptoms, and school adjustment. Specifically, sports participation was positively associated with peer relationships, which in turn were negatively associated with depressive symptoms, and this lower depressive symptoms was further associated with better school adjustment. This pattern of associations is consistent with the basic logic of self-determination theory. According to this theory, the satisfaction of basic psychological needs is considered essential for individuals’ positive development (63). Sports participation, as an activity that involves both skill development and social interaction, may simultaneously address adolescents’ needs for autonomy, competence, and a sense of belonging (64). The fulfillment of the need for belonging could be reflected in increased peer support and improved quality of peer relationships (65). In the context of sports, adolescents may gradually establish a network of mutual trust and emotional support through cooperation, helping each other, and healthy competition. High-quality peer relationships, in turn, may serve as a stable source of social support, which may help buffer the negative emotional impact of daily stress and be associated with a lower risk of depressive symptoms. When depressive symptoms levels are lower, adolescents might be better positioned to engage more positively and with greater intrinsic motivation in school life, which could contribute to enhanced school adjustment. By exploring the interplay among sports participation, peer relationships, and depressive symptoms, these findings highlight the potential value of considering both school- and family-based strategies that support adolescents’ mental health and school adjustment without implying causal certainty.

### 4.6 Practical implications

The findings of this study offer several actionable implications for educational practice. First, given that peer relationships explained 22.53% of the total indirect effect, schools should prioritize structured group sports activities at least three times per week, followed by brief peer-sharing sessions to strengthen social bonding. Second, although the indirect pathway through depressive symptoms was relatively small (2.93%), schools could integrate brief mindfulness or relaxation exercises into physical education classes to help reduce mild depressive symptoms, which may indirectly benefit school adjustment. Third, for only-child adolescents who showed lower peer relationship scores despite higher sports participation teachers should design cooperative, non-competitive activities to reduce social anxiety. Fourth, for students in remote areas where sports facilities are limited, policymakers might consider mobile sports programs or low-cost equipment to increase accessible participation opportunities. These strategies, grounded in the serial mediation mechanism, provide concrete directions for promoting adolescent school adjustment through sports participation.

### 4.7 Research limitations and future directions

This study has several limitations. First, cross-sectional designs cannot establish causal relationships between variables. All reported pathways represent only statistical indirect associations. Future research can use longitudinal follow-up or experimental designs to better test the direction of causality. Second, all variables were measured using self-report scales, which resulted in overly subjective responses. Future research should incorporate objective measures of the variables. Third, although the chain mediation effect was significant, the model included a limited set of variables. Notably, potential confounders such as extraversion, family socioeconomic status (SES), and parenting styles were not measured, which may jointly influence sports participation, peer relationships, and school adjustment. Future studies should incorporate these and other relevant variables to strengthen causal inference and exclude alternative explanations. Last, The sample of this study was drawn entirely from Shandong Province, China, representing a single cultural context. China’s unique family structure, educational system, and sociocultural values may limit the generalizability of the findings. Therefore, caution should be exercised when extending the conclusions to other cultural backgrounds.

## 5 Conclusion

This study used survey data from 8,096 adolescents in Shandong Province to examine the relationships among sports participation, peer relationship, depressive symptoms, and school adjustment. The results showed that sports participation was significantly positively associated with school adjustment. In addition, peer relationships and depressive symptoms each partially explained this association and, together, formed a serial indirect pathway linking sports participation to school adjustment. Furthermore, significant differences in these variables were found across gender, place of residence, and whether the adolescent was an only child.

## Data Availability

The datasets presented in this study can be found in online repositories. The names of the repository/repositories and accession number(s) can be found at: https://www.ncmi.cn/phda/dataDetails.do?id=CSTR:17970.11.A0031.202107.209.V1.0-V2.0.

## References

[1] Lu B Lin L Su X. Global burden of depression or depressive symptoms in children and adolescents: a systematic review and meta-analysis. J Affect Disord. (2024) 354:553–62. doi: 10.1016/j.jad.2024.03.074, 38490591

[2] Li JY Li J Liang JH Qian S Jia RX Wang YQ . Depressive symptoms among children and adolescents in China: a systematic review and Meta-analysis. Med Sci Monit. (2019) 25:7459–70. doi: 10.12659/MSM.916774, 31586039 PMC6792515

[3] Liu C Sun Z Gao Y Chen H Lu T Yan J. The relationship between perceived social support and school adjustment in adolescents: the mediating role of school belongingness and the moderating role of physical activity. Psychol Sch. (2025) 62:1926–36. doi: 10.1002/pits.23444

[4] Zhang LQ Gao HN. Effects of sports on school adaptability, resilience and cell phone addiction tendency of high school students. World J Psychiatry. (2023) 13:563–72. doi: 10.5498/wjp.v13.i8.563, 37701539 PMC10494778

[5] Sun QF Jiao LY zhang GL. The effect of physical activity on social adjustment in adolescents: a serial mediation model and the gender differences. Psychol Res. (2024) 17:375–82. doi: 10.19988/j.cnki.issn.2095-1159.2024.04.011

[6] Kwon J Roh SY Kwon D. Correlation between physical activity and learning concentration, self-management, and interpersonal skills among Korean adolescents. Children (Basel). (2024) 11:1328. doi: 10.3390/children11111328, 39594903 PMC11592538

[7] Jiang XY Chen Z Zhao L zhou JM Ning Z Wang H. Cross-lagged analysis of adolescent peer relationships associated with depression and anxiety. Mod Prev Med. (2025) 52:1986–91. doi: 10.20043/j.cnki.MPM.202501298

[8] Smith AL. Peer relationships in physical activity contexts: a road less traveled in youth sport and exercise psychology research. Psychol Sport Exerc. (2003) 4:25–39. doi: 10.1016/S1469-0292(02)00015-8

[9] Cheng W Jiao L. The mediation effect of peer relation and positive emotion between campus sports atmosphere and teenagers’ subjective well-being. Front Psychol. (2024) 15:1471645. doi: 10.3389/fpsyg.2024.1471645, 39742048 PMC11686361

[10] Pierannunzio D Spinelli A Berchialla P Borraccino A Charrier L Dalmasso P . Physical activity among Italian adolescents: association with life satisfaction, self-rated health and peer relationships. Int J Environ Res Public Health. (2022) 19:4799. doi: 10.3390/ijerph19084799, 35457667 PMC9032550

[11] Livesey D Lum Mow M Toshack T Zheng Y. The relationship between motor performance and peer relations in 9- to 12-year-old children. Child Care Health Dev. (2011) 37:581–8. doi: 10.1111/j.1365-2214.2010.01183.x, 21143269

[12] Liu Y Huebner ES Tian L. Chinese children’s heterogeneous friendship quality trajectories: relations with school adjustment. School Psychol. (2022) 37:410–9. doi: 10.1037/spq0000507, 35797154

[13] Jiménez-Rodríguez T De la Barrera U Schoeps K Valero-Moreno S Montoya-Castilla I. Longitudinal analysis of adolescent adjustment: the role of attachment and emotional competence. Children. (2022) 9:1711. doi: 10.3390/children9111711, 36360439 PMC9689061

[14] Wang S Chong ZY Zhang C Xu W. Longitudinal associations between anxiety and depressive symptoms in adolescence, early adulthood, and old age: cross-lagged panel network analyses. Depress Anxiety. (2024) 2024:6205475. doi: 10.1155/da/6205475, 40226714 PMC11919059

[15] Yu M Mao M Tang C Xing S. Mother-adolescent consistency and discrepancy in perceived maternal psychological control to adolescent depression: exploring the specificity of adolescent gender and age. J Youth Adolescence. (2025) 54:2441–53. doi: 10.1007/s10964-025-02242-4, 40833526

[16] Du Y Cao Y Duan X Zheng W Chen J Qu M. Family structure and depression in Chinese adolescents: the mediating role of resilience. J Affect Disord. (2026) 397:120982. doi: 10.1016/j.jad.2025.120982, 41422957

[17] Trager BM Koning IM Rainosek LM Geurts SM van den Eijnden RJJM Vossen HGM. The longitudinal effects of parent–adolescent digital communication on depression and anxiety symptoms. J Adolesc Health. (2025) 77:610–9. doi: 10.1016/j.jadohealth.2025.05.025, 40838903 PMC13205960

[18] Fu Q Li L Li Q Wang J. The effects of physical activity on the mental health of typically developing children and adolescents: a systematic review and meta-analysis. BMC Public Health. (2025) 25:1514. doi: 10.1186/s12889-025-22690-8, 40269876 PMC12016293

[19] Qian Y Lou H. The buffering effect of physical activity on adolescent psychological stress: a cross-sectional survey and a longitudinal follow-up of Chinese adolescents. BMC Public Health. (2025) 25:3410. doi: 10.1186/s12889-025-24604-0, 41063060 PMC12505626

[20] Noetel M Sanders T Gallardo-Gómez D Taylor P del Pozo Cruz B van den Hoek D . Effect of exercise for depression: systematic review and network meta-analysis of randomised controlled trials. BMJ. (2024) 384:e075847. doi: 10.1136/bmj-2023-075847, 38355154 PMC10870815

[21] Shalayiding S Meng W Wang X Sailike B Jiang T. Symptom network differences in school adjustment and anxiety-depression-stress in adolescents: a gender-based perspective. BMC Public Health. (2024) 24:3189. doi: 10.1186/s12889-024-20718-z, 39551724 PMC11569611

[22] Ryan RM Deci EL. Self-determination theory and the facilitation of intrinsic motivation, social development, and well-being. Am Psychol. (2000) 55:68–78. doi: 10.1037/0003-066X.55.1.68, 11392867

[23] Kowalski KC Crocker PRE Kowalski NP. Convergent validity of the physical activity questionnaire for adolescents. Pediatr Exerc Sci. (1997) 9:342–52. doi: 10.1123/pes.9.4.342

[24] Li X Wang Y Li XT Li DF Sun C Xie MH . Reliability and validity of physical activity questionnaire for adolescents (PAQ-A) in Chinese version. J Beijing Sport Univ. (2015) 38:63–7. doi: 10.19582/j.cnki.11-3785/g8.2015.05.012

[25] Merrell K. Using behavior rating-scales to assess social skills and antisocial-behavior in school settings - development of the school social-behavior scales. Sch Psychol Rev. (1993) 22:115–33. doi: 10.1080/02796015.1993.12085641

[26] Yang Y Gao Y Yi X Hu Y Zhao L Chen L . Does physical activity affect social skills and antisocial behavior? The gender and only child status differences. Front Public Health. (2024) 12:1502998. doi: 10.3389/fpubh.2024.1502998 39624419 PMC11609068

[27] Wu HR Liu PL Meng H. Norm, reliability and validity of children and adolescents’ QOL scale. Chin J Sch Health. (2006) 1:18–21.

[28] Derogatis LR Lipman RS Covi L. SCL-90: an outpatient psychiatric rating scale--preliminary report. Psychopharmacol Bull. (1973) 9:13–28. 4682398

[29] Bai D Wu M Pang Y Liu X. Gender and depression: dual pathways influencing adolescents’ physical activity and psychological wellbeing. Front Public Health. (2025) 13:1663388. doi: 10.3389/fpubh.2025.1663388, 41179749 PMC12575225

[30] Bang H Chang M Kim S. Team and individual sport participation, school belonging, and gender differences in adolescent depression. Child Youth Serv Rev. (2024) 159:107517. doi: 10.1016/j.childyouth.2024.107517

[31] Li M Huang Y Sun M. Exploring the structural links between peer support, psychological resilience, and exercise adherence in adolescents: a multigroup model across gender and educational stages. BMC Public Health. (2025) 25:2300. doi: 10.1186/s12889-025-23308-9, 40610991 PMC12224607

[32] Potter JR Yoon KL. Interpersonal factors, peer relationship stressors, and gender differences in adolescent depression. Curr Psychiatry Rep. (2023) 25:759–67. doi: 10.1007/s11920-023-01465-1, 37773480

[33] Zhao G Xiao L r Chen Y h Zhang M Peng K w Wu H m. Association between physical activity and mental health problems among children and adolescents: a moderated mediation model of emotion regulation and gender. J Affect Disord. (2025) 369:489–98. doi: 10.1016/j.jad.2024.10.041, 39395680

[34] Fine SL Harrison A Rykiel NA Herrera MM. Adolescents’ perceptions of gendered influences on mental health: results from a 13-country qualitative study. J Adolesc Health. (2025) 77:413–20. doi: 10.1016/j.jadohealth.2024.02.036, 40908058

[35] Feiss R Pangelinan MM. Relationships between physical and mental health in adolescents from low-income, rural communities: univariate and multivariate analyses. Int J Environ Res Public Health. (2021) 18:1372. doi: 10.3390/ijerph18041372, 33546117 PMC7913137

[36] Guo X Yang X Mao S. Study on the impact of rural public sports facilities and instructors on residents’ participation in sports activities in China. Front Public Health. (2025) 13:1475321. doi: 10.3389/fpubh.2025.1475321, 40078774 PMC11897029

[37] Yom-Tov E Lekkas D Heinz MV Nguyen T Barr PJ Jacobson NC. Digitally filling the access gap in mental health care: An investigation of the association between rurality and online engagement with validated self-report screens across the United States. J Psychiatr Res. (2022) 157:112–8. doi: 10.1016/j.jpsychires.2022.11.024, 36462251 PMC9898139

[38] Kracht CL Sisson SB. Sibling influence on children’s objectively measured physical activity: a meta-analysis and systematic review. BMJ Open Sport Exerc Med. (2018) 4:e000405. doi: 10.1136/bmjsem-2018-000405, 30364499 PMC6196974

[39] Wang H Verdery AM Margolis R. Sibling availability, sibling sorting, and subjective health among Chinese adults. Demography. (2024) 61:797–827. doi: 10.1215/00703370-11376831, 38814170 PMC11823426

[40] Wikle JS Ackert E Jensen AC. Companionship patterns and emotional states during social interactions for adolescents with and without siblings. J Youth Adolescence. (2019) 48:2190–206. doi: 10.1007/s10964-019-01121-z, 31478120

[41] Owen KB Foley BC Wilhite K Booker B Lonsdale C Reece LJ. Sport participation and academic performance in children and adolescents: a systematic review and Meta-analysis. Med Sci Sports Exerc. (2022) 54:299–306. doi: 10.1249/MSS.0000000000002786, 34559728

[42] Men J Yu Z An W Wang P Hou X Zhang Y . Effects of exercise on cardiorespiratory fitness in children and adolescents with overweight and obesity: a systematic review and meta-analysis of 72 randomized controlled trials. BMC Public Health. (2025) 25:3899. doi: 10.1186/s12889-025-25254-y, 41219861 PMC12607143

[43] Abassi W Ouerghi N Feki M Marsigliante S Bouassida A Knechtle B . Acute effects of exercise on metabolic, inflammatory, and immune markers in adolescent girls with Normal weight or overweight/obesity. Sports (Basel). (2026) 14:24. doi: 10.3390/sports14010024, 41590966 PMC12845669

[44] Liu J Min L Liu R Zhang X Wu M di Q . The effect of exercise on cerebral blood flow and executive function among young adults: a double-blinded randomized controlled trial. Sci Rep. (2023) 13:8269. doi: 10.1038/s41598-023-33063-9, 37217511 PMC10203129

[45] Tomoto T Verma A Kostroske K Tarumi T Patel NR Pasha EP . One-year aerobic exercise increases cerebral blood flow in cognitively normal older adults. J Cereb Blood Flow Metab. (2023) 43:404–18. doi: 10.1177/0271678X221133861, 36250505 PMC9941859

[46] Zhang J Liu Y Zhang X Hu S. How physical activity enhances academic engagement in middle school: a serial mediation model of interpersonal relationships and academic support. Front Psychol. (2025) 16:1632130. doi: 10.3389/fpsyg.2025.1632130, 40861373 PMC12376789

[47] Biddle SJH Ciaccioni S Thomas G Vergeer I. Physical activity and mental health in children and adolescents: an updated review of reviews and an analysis of causality. Psychol Sport Exerc. (2019) 42:146–55. doi: 10.1016/j.psychsport.2018.08.011

[48] Chen R Wang L Wang B Zhou Y. Motivational climate, need satisfaction, self-determined motivation, and physical activity of students in secondary school physical education in China. BMC Public Health. (2020) 20:1687. doi: 10.1186/s12889-020-09750-x, 33172411 PMC7657358

[49] Zhang Z Yu Q Chen Y Zou L Ludyga S Mavilidi M . A dual-process framework for understanding how physical activity enhances academic performance through domain-general and domain-specific executive functions. Educ Psychol Rev. (2025) 37:68. doi: 10.1007/s10648-025-10049-9

[50] Ontiveros N Wiklund CA Ohlis A Ekblom Ö. The role of physical activity in the association between ADHD and emotional dysregulation. J Affect Disord. (2025) 376:68–75. doi: 10.1016/j.jad.2025.01.127, 39889934

[51] Zhao L Lu H Yang Q Zhang D. Intervention effect of a single exercise session on executive function in children and adolescents with attention deficit hyperactivity disorder: a three-level meta-analysis. BMC Pediatr. (2025) 25:519. doi: 10.1186/s12887-025-05846-8, 40604676 PMC12220223

[52] Hu Q Xu C Jiang Y Hu Y Wang Y Ren H . Uncovering the complex effects of socioeconomic status and executive functions on academic achievement: a systematic review and meta-analysis. Educ Psychol Rev. (2025) 37:112. doi: 10.1007/s10648-025-10091-7

[53] Stagnone N Alexander KN Adams KV Dorsch T. Nature’s medicine? The associations of organized youth sport, unstructured physical activity, and land-use recreation with children’s mental health, emotional control, and social well-being. Int J Environ Res Public Health. (2025) 22:1012. doi: 10.3390/ijerph22071012, 40724079 PMC12295727

[54] Zeng Q Wen P Guo K. The impact of physical exercise on adolescents’ social-emotional competence: the chain mediating role of social support and peer relationships. PLoS One. (2025) 20:e0334587. doi: 10.1371/journal.pone.0334587, 41259296 PMC12629442

[55] Bengtsson D Svensson J Wiman V Stenling A Lundkvist E Ivarsson A. Health-related outcomes of youth sport participation: a systematic review and meta-analysis. Int J Behav Nutr Phys Act. (2025) 22:89. doi: 10.1186/s12966-025-01792-x, 40598359 PMC12220085

[56] Allen K Kern ML Vella-Brodrick D Hattie J Waters L. What schools need to know about fostering school belonging: a Meta-analysis. Educ Psychol Rev. (2016) 30:1–34. doi: 10.1007/s10648-016-9389-8, 30311153

[57] Chang CW Yuan R Chen JK. Social support and depression among Chinese adolescents: the mediating roles of self-esteem and self-efficacy. Child Youth Serv Rev. (2018) 88:128–34. doi: 10.1016/j.childyouth.2018.03.001

[58] Tyne WP Fletcher D Paine NJ Stevinson C. Effects of outdoor recreational physical challenges on general self-efficacy: a randomized controlled trial. Psychol Sport Exerc. (2024) 74:102693. doi: 10.1016/j.psychsport.2024.102693, 38960348

[59] Pels F Kleinert J. Loneliness and physical activity: a systematic review. Int Rev Sport Exerc Psychol. (2016) 9:231–60. doi: 10.1080/1750984X.2016.1177849 PMC4706019 26807143

[60] Schumacher A Campisi SC Khalfan AF Merriman K Williams TS Korczak DJ. Cognitive functioning in children and adolescents with depression: a systematic review and meta-analysis. Eur Neuropsychopharmacol. (2024) 79:49–58. doi: 10.1016/j.euroneuro.2023.11.005, 38128461

[61] Choe C Yu S. Longitudinal cross-lagged analysis between depressive symptoms, social withdrawal, self-esteem, and school adaptation in multicultural adolescents. Psychol Belg. (2025) 65:38–53. doi: 10.5334/pb.1310, 39866533 PMC11760225

[62] Meng X Chen Y Rudasill KM Xu L Yu D Harrell DR. The reciprocal relationship between school connectedness and adolescent depressive symptoms: a meta-analytic cross-lagged panel analysis. J Youth Adolescence. (2025) 54:2574–92. doi: 10.1007/s10964-025-02212-w, 40576902

[63] Slemp GR Field JG Ryan RM Forner VW Van Den Broeck A Lewis KJ. Interpersonal supports for basic psychological needs and their relations with motivation, well-being, and performance: a meta-analysis. J Pers Soc Psychol. (2024) 127:1012–37. doi: 10.1037/pspi0000459, 38635183

[64] Mossman LH Slemp GR Lewis KJ Colla RH O’Halloran P. Autonomy support in sport and exercise settings: a systematic review and meta-analysis. Int Rev Sport Exerc Psychol. (2024) 17:540–63. doi: 10.1080/1750984X.2022.2031252

[65] Li M Zheng Y Xie Y Li X. Friendship quality and positive emotional adjustment among boarding adolescents: roles of basic psychological needs satisfaction. Curr Psychol. (2024) 43:6678–90. doi: 10.1007/s12144-023-04802-y

